# Effectiveness of occupational therapy in improving activities of daily living performance in complete cervical tetraplegic patients; A quasi experimental study

**DOI:** 10.12669/pjms.36.2.1002

**Published:** 2020

**Authors:** Aatik Arsh, Zunaira Anwar, Amir Zeb, Syed Muhammad Ilyas

**Affiliations:** 1 Dr. Aatik Arsh, DPT, MSPT. Physical Therapist, Paraplegic Center Peshawar, Peshawar, Pakistan; 2 Zunaira Anwar B.Sc. (Hons.). Occupational Therapist, Paraplegic Center Peshawar, Peshawar, Pakistan; 3 Amir Zeb, PPDPT, MSPT. Senior Physical Therapist, Paraplegic Center Peshawar, Peshawar, Pakistan; 4 Syed Muhammad Ilyas, MSPT. Chief Executive Officer, Paraplegic Center Peshawar, Peshawar, Pakistan

**Keywords:** Cervical, Injury, Occupational therapy, Self-care, Spinal cord

## Abstract

**Objective:**

To determine the effectiveness of Occupational therapy (OT) in improving activities of daily living performance in complete cervical tetraplegic patients.

**Methods:**

A quasi experimental study was conducted at Paraplegic Center Peshawar from May 2018 to March 2019. Seventy two spinal cord injury patients with complete cervical tetraplegia having age 18-60 years were included in the study using non probability convenience sampling technique. A trained Occupational therapist conducted two occupational therapy sessions per day, three times a week on alternative days for a period of six weeks. Self-care portion of Spinal cord injury independence measure (SCIM) was used to collect pre and post data. Data was analyzed using SPSS version 20.

**Results:**

Mean age of the participants was 30.21±13.52.Majority of the participants were (n= 61, 84.7%) male while remaining 11 (15.3%) participants were female. Pre self-care total score was 0.39±0.987 and post self-care total score was 7.17±5.536. There was significant differences (P value <0.05) between pre and post scores of feeding, upper & lower body bathing, upper & lower body dressing, grooming and total self-care scores.

**Conclusion:**

Occupational therapy significantly improves activities of daily living performance in complete cervical tetraplegic spinal cord injury patients.

## INTRODUCTION

Spinal cord injury (SCI) is a devastating injury and is associated with incredible human sufferings and financial costs.^1^ Besides paralysis, bowel and bladder incontinence, sexual dysfunction and other physical sufferings, SCI has profound social, financial and psychological implications.^2^ Though SCI is incurable but rehabilitation strategies aim to minimize complications and maximize independence according to patient functional capabilities.^3^ Occupational therapy (OT) is one of the major pillar of rehabilitation which focuses on improving activities of daily living (ADLs) performance and fine motor activities.^4^

SCI patients with injury at cervical level can’t perform even simple activities such as self eating, dressing and grooming etc. because of impaired upper limb motor functions.^5^ That’s the reason these patients remain bed bounded and remain dependent for the rest of their life.^6^ OT assists these patients in returning to a productive and fulfilling life by enabling them to perform their self-care activities. With the help of adaptive or supportive equipments and through regular upper limb activities, Occupational therapist train cervical SCI patients to perform their ADLs independently.^5^ Besides optimization of functional independence, OT training also helps in improving community integration and prevention of secondary complications.^7^

Majority of published research studies related to cervical SCI patients focused either on hand surgery or increasing physical capacity in these patients.^5^,^7^-^9^ Nevertheless, these do not automatically lead to improved self-care activities. In Pakistan, OT is commonly used in clinical practice to improve upper limb activities in cervical SCI patients and majority of rehabilitation centers dealing with SCI, have dedicated OT departments, however limited literature is available regarding effectiveness of OT in improving self-care activities in cervical SCI patients. Therefore, there was a dire need to conduct current study in order to determine effectiveness of OT in improving ADL performance in complete cervical tetraplegic patients.

## METHODS

A quasi experimental study was conducted at Paraplegic Center Peshawar from May 2018 to March 2019. Ethical approval was obtained from Ethics committee of Paraplegic Centre Peshawar (No. DIR/PCP-EB/00, dated May 6, 2019).

Seventy two SCI patients with complete cervical tetraplegia having age 18-60 years were included in the study using non probability convenience sampling technique. The American Spinal Injury Association (ASIA) classification system was used to confirm whether the patient has complete (ASIA A) or incomplete (ASIA B, C, D or E) injury. Cervical tetraplegics with injury above C4 or having injury due to non traumatic causes were excluded. SCI patients with complications e.g. deep venous thrombosis, pressure sores etc and those with other associated injuries such as traumatic brain injury and fractures etc. were also excluded. Informed consent was taken from all the eligible participants who were willing to participate in the study. Subjects underwent a full neurological and cognitive assessment before commencement of study.

A trained Occupational therapist conducted two OT sessions per day, three times a week on alternative days for a period of six weeks.OT assistants were available to assist in conducting the sessions. Each session lasted for 45 minutes to one hour and consisted of following activities: Upper limb strengthening exercises such as sanding activities, weight lifting and resistance training etc., fine motor activities such as cloth peg activity, box and block activity and peg board activity etc., and ADL training such as self eating, bathing, dressing and grooming with adaptive or supportive equipments. Moreover, tenodesis grip training for grip improvement with tapping, bandaging and splinting were also performed. Self-care portion of Spinal cord injury independence measure (SCIM)^10^ was used to collect pre and post data. Self-care portion of SCIM has questions related to ADLs such as feeding, bathing, dressing and grooming.

Data was analyzed using SPSS version 20. Paired sample T- test was used to compare pre and post interventional scores. P value <0.05 was considered significant.

## RESULTS

Mean age of the participants was 30.21±13.52.Majority of the participants were (n= 61, 84.7%) male while remaining 11 (15.3%) participants were female. Forty four (61.1%) participants were married and 28 (38.9%) were single. Twenty three (31.9%) participants were illiterate while the remaining 49 (68.1%) had different level of education from primary to post graduation.

Pre feeding score was 0.25±0.550 while post feeding score was 2.07±0.793. Similarly, pre self-care total score was 0.39±0.987 and post self-care total score was 7.17±5.536. There was significant differences (P value <0.05) between pre and post scores of feeding, upper & lower body bathing, upper & lower body dressing, grooming and total self-care scores (Table-I).

**Table-I:** Pre and post intervention scores of self-care component of SCIM.

	Mean± SD	P-value
Pre Feeding score	0.25±0.550	<0.001
Post Feeding score	2.07±0.793	
Pre Upper body bathing score	0.01±0.118	<0.001
Post Upper body bathing score	1.06±1.073	
Pre Lower body bathing score	0.00±0.000	<0.001
Post Lower body bathing score	0.53±0.855	
Pre Upper body dressing score	0.01±0.118	<0.001
Post Upper body dressing score	1.31±1.360	
Pre Lower body dressing score	0.00±0.000	<0.001
Post Lower body dressing score	0.62±1.027	
Pre Grooming score	0.11±0.430	<0.001
Post Grooming score	1.60±1.030	
Pre Total score	0.39±0.987	<0.001
Post Total score	7.17±5.536	

## DISCUSSION

Occupational therapy is a main part of rehabilitation process of SCI patients which deals with problems related to self-care and ADLs.^11^ SCI patients in general and cervical tetraplegics in particular have difficulties in performing ADLs.^12^ OT interventions focusing on self-care activities are critical for cervical tetraplegics to live independently and to minimize the burden of care.^13^ That’s why independence in self-care activities is one of the most important goal of Occupational Therapists in rehabilitation settings.^8^ To achieve this goal, Occupational Therapists apply a wide range of OT interventions including but not limited to therapeutic exercises, splinting, adoptive/supportive equipments training (eating aids, writing aids, typing aids, cell phone holders), ADL training (dressing, combing, washing, bathing etc) and fine motor activities of hand.^3^,^9^,^14^ Despite the fact that these OT activities are commonly used in clinical practice but literature regarding their effectiveness is scarce. Therefore current quasi experimental study was conducted to report effectiveness of OT interventions in improving self-care activities in cervical SCI patients.

Almost 85% participants in current study were male and majority of the participants were of young age. These results were consistent with other studies conducted about SCI in Pakistan.^6^,^15^ In Pakistan, women are relatively safer compared to men as majority of the former population are less exposed to outdoor dangers. That’s why SCI is less common in female. Similarly, young population is more active in their 2^nd^ and 3^rd^ decade of life, therefore traumatic SCI is more common in young age. Previous studies have reported that cervical SCI are less common as compared to thoracic and lumbar injuries,^15^,^16^ however exact statistics about cervical tetraplegic SCI patients in Pakistan are not available. Literature search also revealed that there is limited literature available regarding rehabilitation outcomes in SCI patients in Pakistani settings^2^,^17^ while there was no published research study which reported OT outcomes in SCI patients in Pakistan.

Results of current study showed that OT interventions significantly improve self-care activities such as feeding, bathing, dressing and grooming etc in cervical tetraplegic patients. Previous studies also reported that OT focused on ADL training and self-care activities can improve functional independence and can help these patients to achieve good rehabilitation outcomes.^18^,^19^ Compared to paraplegic SCI patients whose only lower limbs are paralyzed, cervical SCI patients has impaired lower as well as upper limb movements. That is the reason that cervical tetraplegic patients have generally lower rehabilitation outcomes as compared to paraplegic patients. Similarly, patients with incomplete SCI has good prognosis and rehabilitation outcomes as compared to complete SCI patients.^20^ Because current study included only complete cervical tetraplegic SCI patients, that’s why rehabilitation outcomes reported in current study were lower as compared to studies which reported rehabilitation outcomes either in paraplegic patients or those who also included incomplete SCI patients.^7^,^20^-^22^Current study is one of the very first study which has reported effectiveness of OT interventions in improving self-care activities in cervical tetraplegic patients in Pakistan.,

### Limitations of the study

Firstly, current study only investigated outcomes of OT in improving ADLs while rehabilitation not only emphasized on ADLs but also includes mobility training, wheel chair maneuverability, bowel & bladder management and much more. Secondly, because current study was conducted in clinical settings therefore it was not possible to control confounding variables. Thirdly, current study was conducted in a single rehabilitation center, due to which results of current study can’t be generalized. Moreover, due to lack of control group in current study, it was not possible to compare outcomes between groups.

It is recommended to conduct large multicenter clinical trials in order to truly determine rehabilitation outcomes in SCI patients in Pakistani settings. In addition, there is a dire need to develop clinical guidelines for rehabilitation of SCI patients which not only address the individual needs of SCI patients but also take into accounts the specific cultural, geographical, economical, and religious factors prevailing in Pakistani society.

## CONCLUSION

OT significantly improves ADL performance and self-care such as feeding, bathing, dressing and grooming in complete cervical tetraplegic SCI patients. OT should be an integral part of rehabilitation settings which deals with SCI patients.

### Authors’ Contributions:

**AA:** Literature search and literature review, Analysis & interpretation of data, drafting the manuscript, Critical revision, is responsible for integrity of the study.

**ZA:** Concept and study design, Acquisition of data, Literature search and literature review.

**AZ:** Acquisition of data, drafting the manuscript

**SM:** Analysis & interpretation of data, Critical revision, final approval of the version to be published.
